# Comparison of Short‐Term Outcomes of Intracorporeal Anastomosis in Robot‐Assisted colon Resection and Laparoscopic colon Resection: A Single‐Center Study and Systematic Review and meta‐Analysis

**DOI:** 10.1111/ases.70366

**Published:** 2026-08-16

**Authors:** Yusuke Tomita, Kohei Shigeta, Yuzuko Momose, Masayoshi Monno, Satoru Morita, Koji Okabayashi, Yuko Kitagawa

**Affiliations:** ^1^ Department of Surgery Keio University School of Medicine Tokyo Japan

**Keywords:** colon cancer, intracorporeal anastomosis, meta‐analysis

## Abstract

**Background:**

This study evaluates short‐term outcomes of robot‐assisted (Rt) and laparoscopic (Lap) intracorporeal anastomosis (IA) in colon resection using an exploratory single‐center retrospective cohort and a systematic review and meta‐analysis conducted in accordance with PRISMA guidelines.

**Methods:**

Patients with colon cancer who underwent laparoscopic or robotic surgery at our institution from 2015 to 2024 were retrospectively enrolled. Additionally, a meta‐analysis was conducted using PubMed, Web of Science, Scopus, and the Cochrane Library to identify studies comparing Rt‐IA and Lap‐IA. Short‐term outcomes were statistically compared between the two groups.

**Results:**

A total of 77 patients were included (Rt‐IA 26, Lap‐IA 51). Intraoperative blood loss was significantly lower in the Rt‐IA group (median, 5 mL vs. 10 mL; *p* = 0.001). No statistically significant differences were detected in major postoperative complications or most recovery‐related outcomes. For the meta‐analysis, 11 studies (1 prospective and 10 retrospective) met the inclusion criteria. After adding our institutional data, the pooled analysis showed that operative time was significantly longer in the Rt‐IA group (MD, 54.02 min; 95% CI, 34.18–73.86; *I* ^2^ = 95.4%), whereas the Rt‐IA group demonstrated significantly less blood loss (MD, −14.33 mL; 95% CI: −27.04 to −1.63; *I* ^2^ = 19.3%).

**Conclusions:**

The results of our institutional analysis together with meta‐analysis suggest that Rt‐IA may be associated with longer operative time but slightly lower blood loss than Lap‐IA. These findings suggest that Rt‐IA may be a feasible alternative to Lap‐IA; however, the results should be interpreted with caution given the limitations of the available evidence.

## 1 Introduction

The use of intracorporeal anastomosis (IA) has increased in minimally invasive colon surgery as surgeons seek to minimize bowel manipulation and improve procedural flexibility [1, 2, 3, 4, 5, 6, 7]. Compared with extracorporeal anastomosis (EA), IA allows for greater freedom in specimen extraction site selection and may contribute to more stable postoperative recovery [3, 4, 5, 6, 7]. The introduction of robotic platforms has further expanded the application of IA by providing enhanced instrument articulation and a stable three‐dimensional operative view [8, 9, 10]. These technical features are expected to facilitate intracorporeal suturing and stapling; however, whether such advantages translate into measurable improvements in short‐term clinical outcomes remains unclear. Previous comparative studies between robot‐assisted IA (Rt‐IA) and laparoscopic IA (Lap‐IA) have reported inconsistent findings. While prolonged operative time with robotic surgery is frequently described, its impact on perioperative outcomes such as complications and recovery remains inconclusive [11, 12]. Because obesity rates are relatively low and colorectal procedures are highly standardized in Japan, perioperative outcomes associated with Rt‐IA may differ from those reported in Western cohorts [4]. In addition, heterogeneity in patient populations and variations in anastomotic techniques across studies may obscure potential differences between the two approaches. In this context, evaluation within a standardized surgical setting may provide additional insight. At our institution, IA has been consistently performed using a functional end‐to‐end anastomosis (FEEA), allowing comparison under relatively uniform technical conditions. Notably, most previous studies have predominantly employed side‐to‐side isoperistaltic anastomosis, whereas data under a standardized FEEA approach remain limited.

Therefore, the present study aimed to compare short‐term outcomes between Rt‐IA and Lap‐IA using a single‐center cohort with a standardized anastomotic approach. In addition, an updated meta‐analysis was conducted to position our findings within the existing body of evidence.

## 2 Materials and Methods

### 2.1 Study Design and Patient Selection

A retrospective cohort study was conducted at our institution to compare short‐term outcomes between Rt‐IA and Lap‐IA. Consecutive patients who underwent minimally invasive colon resection with IA between January 2015 and December 2024 were reviewed. Eligible patients had histologically confirmed colon cancer located from the cecum to the sigmoid colon and underwent IA reconstruction. To minimize technical variability, all anastomoses were performed using a FEEA technique. Patients with inflammatory bowel disease, synchronous malignancies, or those who underwent EA were excluded. There were no strictly prespecified criteria for selecting intracorporeal anastomosis or the robotic approach. In general, the choice of IA and surgical platform was based on patient and tumor characteristics, surgeon preference, and platform availability. Because robotic surgery was introduced in 2018 and Rt‐IA in 2023, differences in study period and perioperative management may have influenced the results. All operations were performed or supervised by two experienced colorectal surgeons who had surpassed the learning curve for robotic surgery (> 40 cases). This study was approved by the ethics committee of our institution (20150051). Because of the retrospective nature of the study, the requirement for written informed consent was waived, and information regarding the study was disclosed on the institutional website with an opt‐out option. This study was conducted in accordance with the Declaration of Helsinki.

### 2.2 Surgical Technique: Intracorporeal FEEA

IA was performed using a standardized FEEA technique in all cases. After resection of the colon, the proximal and distal bowel segments were aligned in an isoperistaltic orientation. Small enterotomies were created on the antimesenteric borders of each segment. A linear stapler was inserted through the enterotomies to create a side‐to‐side anastomosis. The common enterotomy was then closed using an additional linear stapler, resulting in a FEEA configuration. Care was taken to ensure adequate blood supply and to avoid tension at the anastomotic site. The procedure was performed intracorporeally under laparoscopic or robotic assistance, depending on the surgical approach. The anastomotic technique was standardized across both groups to minimize technical variability.

### 2.3 Clinical Outcomes

Patient demographics and perioperative data were collected from the medical records. Variables included age, sex, body mass index (BMI), and American Society of Anesthesiologists classification. The primary outcomes were intraoperative and short‐term postoperative results. Operative variables included operative time, estimated blood loss, and conversion to open surgery. Postoperative outcomes included complications (anastomotic leakage, surgical site infection, postoperative hemorrhage, and ileus), time to first flatus, time to oral intake, and length of hospital stay. Postoperative complications were assessed using the Clavien‐Dindo classification, and major complications were defined as Grade III or higher. Anastomotic leakage was defined based on clinical presentation and/or radiological findings. Postoperative ileus was defined as intolerance to oral intake or the requirement for nasogastric decompression. Outcomes were evaluated within 30 days after surgery.

### 2.4 Meta‐Analysis: Search Strategy and Study Selection

This study was conducted in accordance with the Preferred Reporting Items for Systematic Reviews and Meta‐Analyses (PRISMA) guidelines. For the meta‐analysis, a systematic literature search of PubMed, Web of Science, Scopus, and the Cochrane Library was performed from database inception to December 2024. The search strategy incorporated Medical Subject Headings (MeSH) and free‐text terms in PubMed and free‐text terms in the other databases. Search terms covered concepts related to intracorporeal anastomosis, robotic and laparoscopic surgery, and colectomy. Additional details of the full search strategy, including search fields and filters, are provided in the Supporting Information.

The inclusion criteria were as follows: (1) studies involving colon resection and (2) comparative study designs evaluating short‐term outcomes between Rt‐IA and Lap‐IA. Exclusion criteria were as follows: (1) incomplete data, (2) duplicate publications, and (3) studies including patients with concomitant malignancies. Two authors independently screened titles, abstracts, and full texts. Disagreements were resolved by discussion and consensus. For studies that included additional comparator groups (e.g., laparoscopic EA), only separately reported outcome data from the Rt‐IA and Lap‐IA groups were extracted for the present meta‐analysis.

### 2.5 Statistical Analysis: Single‐Institution Cohort

Categorical variables were compared using the chi‐square test or Fisher's exact test, as appropriate. Continuous variables are expressed as medians [interquartile ranges (IQRs)] and compared using the Mann–Whitney U test for non‐normally distributed data. A *p* < 0.05 was considered statistically significant. All statistical analyses of the institutional cohort were performed using Stata (version 14; StataCorp LP, College Station, TX, USA).

### 2.6 Statistical Analysis

Meta‐Analysis.

For the quantitative synthesis, statistical pooling was performed with EZR (Saitama Medical Center, Jichi Medical University, Saitama, Japan), which provides a graphical interface for R [13]. Summary estimates were generated with a random‐effects approach in view of anticipated heterogeneity across studies. Binary outcomes were expressed as odds ratios and continuous outcomes as mean differences, each with corresponding 95% confidence intervals. Where studies reported medians and dispersion measures instead of means and standard deviations, these values were converted using previously described methods [14, 15]. Small‐study effects were explored through funnel plot inspection together with Egger's test. Study quality was appraised with the Newcastle‐Ottawa Scale, and certainty of evidence was judged using the GRADE approach.

## 3 Results

### 3.1 Single‐Institution Data

**FIGURE 1:**
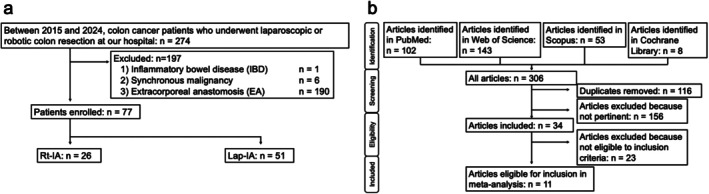
(a) Flow chart of patient inclusion. IA: Intracorporeal anastomosis. (b) The PRISMA diagram. PRISMA, Preferred Reporting Items for Systematic Reviews and Meta‐Analyses.

A total of 274 patients who underwent laparoscopic and robotic colon resection between January 2015 and December 2024 were initially identified. After application of the exclusion criteria, 197 patients were excluded: one due to concomitant inflammatory bowel disease, six due to synchronous malignancy, and 190 who underwent EA. Following application of the exclusion criteria, 77 patients were eligible for the final comparison: 26 treated with Rt‐IA and 51 with Lap‐IA (Figure 1a). Baseline features are detailed in Table 1. Aside from tumor location, which differed between the groups (*p* = 0.003), the clinical background was largely similar.

**TABLE 1:** Baseline characteristics.

	Rt‐IA^a^ *n* = 26	Lap‐IA^b^ *n* = 51	*p*
Sex			0.17
Male (%)	11 (42)	30 (59)	
Female (%)	15 (58)	21 (41)	
Age (median) (IQR^c^)	75 (61–80)	70 (58–79)	0.51
BMI^d^ (kg/m^2^) (median) (IQR)	21.0 (19.3–23.6)	22.7 (20.1–25.1)	0.09
ASA^e^			0.52
I (%)	19 (73)	32 (63)	
II (%)	5 (19)	16 (31)	
III (%)	2 (8)	3 (6)	
Tumor location			0.003
Cecum (%)	4 (15)	16 (31)	
Ascending colon (%)	14 (54)	27 (53)	
Hepatic flexure (%)	0	0	
Transverse colon (%)	1 (4)	6 (12)	
Splenic flexure (%)	1 (4)	0	
Descending colon (%)	4 (15)	1 (2)	
Sigmoid colon (%)	2 (8)	1 (2)	

^a^ Robot‐assisted intracorporeal anastomosis.

^b^ Laparoscopic intracorporeal anastomosis.

^c^ Interquartile range.

^d^ Body mass index.

^e^ American society of anesthesiologists.

Table 2 summarizes the perioperative results. Blood loss and time to first flatus were lower in the Rt‐IA group (blood loss *p* = 0.001; time to first flatus *p* = 0.007), whereas no clear between‐group differences were detected for operative duration, other recovery parameters, postoperative complications, or length of stay. Conversion to open surgery was not required in either group. Pathological findings were similar between the two approaches (Table S1).

**TABLE 2:** Operative and postoperative outcomes.

	Rt‐IA^a^ *n* = 26	Lap‐IA^b^ *n* = 51	*p*
Operative time (min) (median) (IQR^c^)	261 (211–305)	267 (233–287)	0.99
Blood loss (ml) (median) (IQR)	5 (5–5)	10 (5–20)	0.001
Postoperative complications (%)	2 (8)	12 (24)	0.08
Anastomotic leakage (%)	1 (4)	3 (6)	0.47
Surgical site infection (%)	1 (4)	3 (6)	0.47
Postoperative hemorrhage (%)	0	2 (4)	0.53
Postoperative ileus (%)	0	4 (8)	0.33
Clavien‐dindo classification			0.13
I (%)	1 (4)	5 (10)	
II (%)	0	4 (8)	
III (%)	0	3 (6)	
IV (%)	1 (4)	0	
V (%)	0	0	
Bowel preparation			0.001
Mechanical preparation only (%)	0	42 (82)	
Mechanical preparation + oral antibiotic (%)	26 (100)	9 (18)	
Time to first flatus (days) (median) (IQR)	2 (1–2)	3 (2–4)	0.007
Time to first stool (days) (median) (IQR)	4 (3–5)	4 (3–5)	0.49
Time to first oral intake (days) (median) (IQR)	4 (3–5)	3 (2–5)	0.09
Length of hospital stay (days) (median) (IQR)	9 (9–10)	7 (5–14)	0.26
Open conversion	0	0	

^a^ Robot‐assisted intracorporeal anastomosis.

^b^ Laparoscopic intracorporeal anastomosis.

^c^ Interquartile range.

### 3.2 Meta‐Analysis

A total of 306 records were identified through database searching. After removal of 116 duplicates, 190 records underwent title and abstract screening. Of these, 156 records were excluded, and 34 full‐text articles were assessed for eligibility. Following full‐text review, 23 articles were excluded, leaving 11 studies for inclusion in the final analysis [11, 12, 16, 17, 18, 19, 20, 21, 22, 23, 24] (Figure 1b). The included literature consisted of one prospective cohort study and 10 retrospective studies published between 2005 and 2024. Study characteristics and perioperative outcomes are summarized in Tables S2–S4.

**FIGURE 2:**
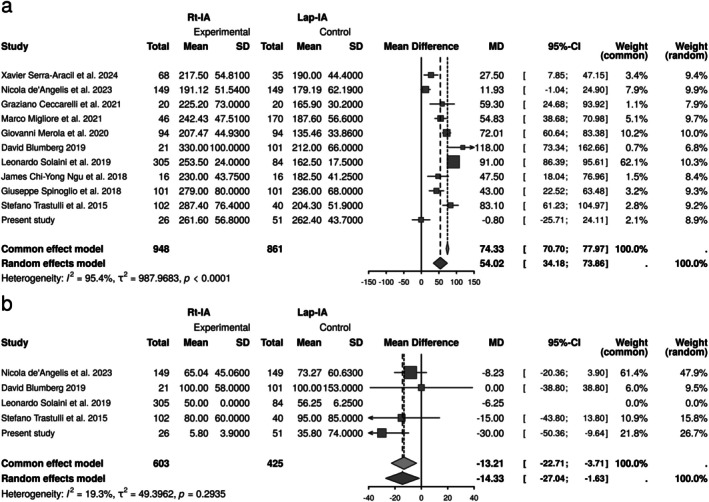
(a) Forest plot of operative time. (b) Forest plot of blood loss.

In total, data from 1909 patients (998 Rt‐IA and 911 Lap‐IA), including the present cohort, were pooled for analysis. The pooled analysis showed that the Rt‐IA group had a significantly longer operative time (MD, 54.02 min; 95% CI, 34.18–73.86; *I* ^2^ = 95.4%) and lower estimated blood loss (MD, −14.33 mL; 95% CI: −27.04 to −1.63; *I* ^*2*^ = 19.3%) than the Lap‐IA group (Figure 2a,b). However, no significant differences were observed between the two groups in rates of anastomotic leakage, surgical site infection, postoperative ileus, postoperative hemorrhage, conversion to open surgery, or recovery‐related outcomes (Figures S1–S8).

**FIGURE 3:**
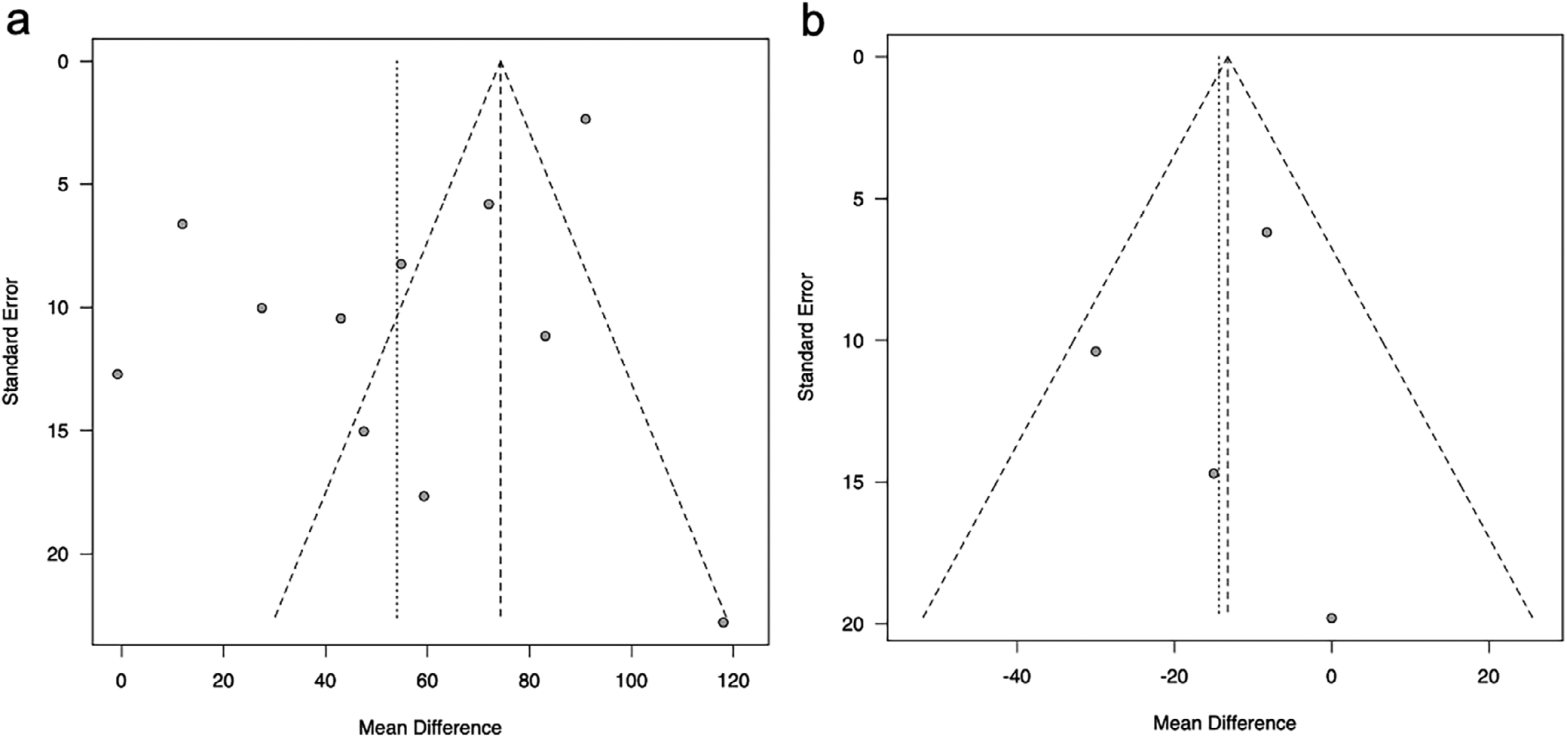
(a) Funnel plot for operative time. (b) Funnel plot for blood loss.

Assessment of publication bias for the primary outcomes revealed asymmetry in the funnel plot for operative time by Egger's test (*p* = 0.03, Figure 3a). In contrast, funnel plot for estimated blood loss was symmetrical, and no significant publication bias was detected (*p* = 0.31, Figure 3b). According to the GRADE framework, the certainty of evidence was low or very low for all evaluated outcomes, whereas study quality based on the Newcastle‐Ottawa Scale was generally moderate to high (Table 3 and Table S5).

**TABLE 3:** GRADE assessment of the certainty of evidence.

Certainty assessment							№ of patients		Effect		Certainty
№ of studies	Study design	Risk of bias	Inconsistency	Indirectness	Imprecision	Other considerations	Rt‐IA	Lap‐IA	Relative (95% CI)	Absolute (95% CI)	
Operative time											
11	Non‐randomized studies	Serious^a^	Very serious^b^	Not serious	Not serious	Publication bias strongly suspected	948	861	—	MD 54.02 (34.18 to 73.86)	⨁◯◯◯ Very low^a^, ^b^
blood loss											
5	Non‐randomized studies	Serious^a^	Not serious	Not serious	Serious^c^	None	603	425	—	MD −14.33 (−27.04 to −1.63)	⨁⨁◯◯ Low^a^, ^c^

Abbreviations: CI: confidence interval; MD: mean difference.

^a^ Most included studies were retrospective observational studies; therefore confounding and selection bias cannot be excluded.

^b^ Considerable heterogeneity was observed (*I* ^2^ = 95.4%), indicating large variation in effect estimates across studies.

^c^ Although statistically significant, the effect size was small (MD −14 mL), and the clinical importance of this difference is uncertain.

## 4 Discussion

This combined analysis of institutional data and pooled evidence suggests that Rt‐IA may be associated with longer operative time and slightly lower estimated blood loss compared with Lap‐IA. However, these findings should be interpreted with caution, as the institutional cohort was small and subject to potential confounding. No consistent differences were observed in postoperative morbidity, and recovery‐related outcomes were largely comparable overall.

Our institutional cohort, integrated with the updated meta‐analysis, supports the feasibility of Rt‐IA as an alternative to Lap‐IA. However, the observed difference in blood loss was small in magnitude, and its clinical relevance remains uncertain. This finding may reflect differences in visualization, instrument articulation, or operative workflow associated with the robotic platform; however, the present study was not designed to determine the underlying mechanism.

Importantly, the institutional data should be regarded as exploratory and descriptive. The sample size was insufficient to adequately evaluate low‐frequency outcomes such as anastomotic leakage or postoperative complications, and continuous variables such as blood loss may be influenced by distributional variability. Furthermore, the relatively small proportion of IA cases over the study period raises the possibility of selection bias. There were no strictly prespecified criteria for selecting intracorporeal anastomosis or the robotic approach. In general, the choice of IA and surgical platform was based on patient and tumor characteristics, surgeon preference, and platform availability.

In addition, the long study period introduces potential chronological bias. Because robotic surgery was implemented later in our institutional experience, patients undergoing Rt‐IA may disproportionately represent a later phase of surgical practice, during which technical proficiency, perioperative management, and institutional protocols may have improved. As a result, the observed differences between groups, including reduced blood loss, may partially reflect temporal improvements rather than intrinsic advantages of the robotic platform.

In contrast to the pooled analysis and several previous reports, no significant difference in operative time was observed between Rt‐IA and Lap‐IA [11, 12, 16, 18, 19, 20, 23, 24]. This discrepancy may be related to the relatively low BMI of the study population, the standardized perioperative and operative environment, and the experience of operating surgeons at our center. However, given the small sample size and potential confounding factors, this interpretation remains speculative.

The meta‐analysis component also has inherent methodological limitations. The included studies were predominantly retrospective and heterogeneous with respect to patient selection, surgical technique, surgeon experience, and perioperative management. Although a random‐effects model was used, the substantial heterogeneity observed for operative time (*I* ^2^ = 95.4%) suggests that pooled estimates should be interpreted as descriptive rather than definitive. Furthermore, publication bias was detected for operative time, indicating that the available evidence may be subject to reporting bias. Therefore, concordance between the institutional cohort and the meta‐analysis should not be interpreted as independent validation, but may instead reflect shared limitations inherent to retrospective study designs.

Regarding anastomotic techniques, several methods are commonly used, including side‐to‐side isoperistaltic anastomosis, FEEA, delta‐shaped anastomosis, and end‐to‐end anastomosis [7]. Side‐to‐side isoperistaltic anastomosis, represented by the Overlap method, is currently the most widely used technique for intestinal anastomosis [12, 17]. In the present study, we exclusively performed FEEA in our cohort. FEEA offers advantages such as shorter anastomotic time and technical simplicity, whereas delta‐shaped anastomosis is characterized by reduced tension on the intestinal tract during anastomosis [25, 26]. Our preference for this method stems from our extensive experience with it in extracorporeal procedures, which facilitated a smooth transition to the IA without a steep learning curve. By adopting a standardized approach, we aimed to ensure consistent anastomotic quality regardless of the operating surgeon. However, most studies included in the meta‐analysis employed different anastomotic techniques, which may have contributed to between‐study heterogeneity. The present study was not designed to compare outcomes across different IA techniques. Finally, important outcomes such as cost‐effectiveness, learning curve burden, and long‐term oncological results were not evaluated in this study. These factors are essential when assessing the overall value of robotic surgery and should be addressed in future research.

In conclusion, Rt‐IA may be a feasible alternative to Lap‐IA in colon resection, with broadly comparable short‐term outcomes. Although Rt‐IA may be associated with slightly lower estimated blood loss, the absolute difference is small, and its clinical significance remains uncertain. Given the limitations of the available evidence, including small sample size, retrospective design, potential confounding, and heterogeneity across studies, these findings should be interpreted as hypothesis‐generating. Further large‐scale prospective studies are required to clarify the true clinical value of Rt‐IA.

## Author Contributions

All authors contributed the study conception and design. Material preparation and data collection were performed by Yusuke Tomita and Yuzuko Momose. Analysis was performed by Yusuke Tomita. The first draft of the manuscript was written by Yusuke Tomita and Kohei Shigeta. All authors commented on previous versions of the manuscript. All authors read and approved the final manuscript.

## Funding

The authors have nothing to report.

## Disclosure

Dr. Kitagawa reports grants and personal fees from Chugai Pharmaceutical Co. Ltd. and Taiho Pharmaceutical Co. Ltd. and grants from Asahi KASEI Co. Ltd., Otsuka Pharmaceutical Co. Ltd., Tsumura & Co., Ono Pharmaceutical Co. Ltd., Takeda Pharmaceutical Co. Ltd., Otsuka Pharmaceutical Factory Inc., Eisai Co. Ltd., and Bayer Yakuhin Ltd. outside of the submitted work.

## Conflicts of Interest

The authors declare no conflicts of interest.

## Supporting information


**Data S1:** ases70366‐sup‐0001‐Supinfo.docx.
**Table S1:** Histopathological outcomes.
**Table S2:** Baseline characteristics of the study population.
**Table S3:** Surgical outcomes of the study population.
**Table S4:** Surgical outcomes of the study population.
**Table S5:** Quality assessment of included studies using the Newcastle‐Ottawa Scale.
**Figure S1:** The meta‐analysis for time to first oral feeding.
**Figure S2:** The meta‐analysis for time to first flatus.
**Figure S3:** The meta‐analysis for length of hospital stay.
**Figure S4:** The meta‐analysis for open conversion.
**Figure S5:** The meta‐analysis for anastomotic leakage.
**Figure S6:** The meta‐analysis for surgical site infection.
**Figure S7:** The meta‐analysis for postoperative ileus.
**Figure S8:** The meta‐analysis for postoperative hemorrhage.

## Data Availability

The data that support the findings of this study are available on request from the corresponding author. The data are not publicly available due to privacy or ethical restrictions.
