# Supplementary material for: Comparison of Short‐Term Outcomes of Intracorporeal Anastomosis in Robot‐Assisted colon Resection and Laparoscopic colon Resection: A Single‐Center Study and Systematic Review and meta‐Analysis
Source: Asian J Endosc Surg. 2026 Aug 16;19(1):e70366. doi: 10.1111/ases.70366 (PMC13477841; doi:10.1111/ases.70366)
Supplement: Supplementary file 1 [file ASES-19-e70366-s001.docx]

Supplemental Table 1 Histopathological outcomes

|  | Rt-IA^a^  n = 26 |  | Lap-IA^b^  n = 51 |  | *P value* |
| --- | --- | --- | --- | --- | --- |
|  |  |  |  |  |  |
| pStage |  |  |  |  | 0.34 |
| I (%) | 13 (50) |  | 22 (43) |  |  |
| II (%) | 2 (8) |  | 12 (24) |  |  |
| III (%) | 8 (30) |  | 14 (27) |  |  |
| IV (%) | 3 (12) |  | 3 (6) |  |  |
| pT stage |  |  |  |  | 0.41 |
| 1 (%) | 11 (42) |  | 18 (35) |  |  |
| 2 (%) | 6 (23) |  | 6 (12) |  |  |
| 3 (%) | 7 (27) |  | 21 (41) |  |  |
| 4 (%) | 2 (8) |  | 6 (12) |  |  |
| pN stage |  |  |  |  | 0.44 |
| 0 (%) | 16 (62) |  | 34 (67) |  |  |
| 1 (%) | 9 (35) |  | 12 (23) |  |  |
| 2 (%) | 1 (3) |  | 5 (10) |  |  |
| Histopathological type |  |  |  |  | 0.53 |
| tub1, tub2 (%) | 25 (96) |  | 47 (92) |  |  |
| por, sig, muc (%) | 1 (4) |  | 4 (8) |  |  |
| Number of lymph nodes retrieved (median) (IQR^c^) | 23 (14-31) |  | 28 (19-38) |  | 0.06 |
| Ly+ (%) | 7 (27) |  | 25 (49) |  | 0.15 |
| V+ (%) | 15 (58) |  | 22 (43) |  | 0.19 |

^a^Robot-assisted intracorporeal anastomosis

^b^Laparoscopic intracorporeal anastomosis

^c^Interquartile range

Supplemental Table 2 Baseline characteristics of the study population

| First Author | Publication year | Study type | Study period | Sample size | | Sex (male/female) | | Age (mean±SD^a^) | | BMI^b^, kg/m^2^ (mean ± SD) | | ASA^c^>III (n (%)) | |
| --- | --- | --- | --- | --- | --- | --- | --- | --- | --- | --- | --- | --- | --- |
|  |  |  |  | Rt-IA^d^ | Lap-IA^e^ | Rt-IA | Lap-IA | Rt-IA | Lap-IA | Rt-IA | Lap-IA | Rt-IA | Lap-IA |
| Serra-Aracil | 2024 | Prospective | 2020-2023 | 68 | 35 | 36/32 | 19/16 | 71±11.11 | 69±11.85 | 27.57±5.75 | 26.22±4.69 | 27 (39.7) | 18 (51.4) |
| Angelis | 2023 | retrospective | 2014-2020 | 149 | 149 | 77/72 | 73/76 | 70.72±10.99 | 71.43±12.79 | 26.99±3.92 | 26.44±4.84 | 4 (2.7) | 3 (2) |
| Ceccarelli | 2021 | retrospective | 2014-2019 | 20 | 20 | 14/6 | 13/7 | 70.6±9.9 | 74.6±13.8 | 23.0±2.4 | 24.1±2.9 | 7 (35) | 6 (30) |
| Migliore | 2021 | retrospective | 2010-2018 | 46 | 170 | 22/24 | 96/74 | 68.70±9.2 | 71.92±10.1 | 26.05±4.05 | 25.52±4.07 | 15 (32.6) | 63 (37.1) |
| Merola | 2020 | retrospective | 2012-2017 | 94 | 94 | 60/34 | 61/33 | 69.41±10.31 | 72.09±9.54 | 26.94±4.61 | 27.97±5.73 | 37 (39.4) | 31 (33) |
| Blumberg | 2019 | retrospective | 2003-2016 | 21 | 101 | 7/14 | 50/51 | 65±10 | 68±12 | 30±7 | 28±7 | 15 (71) | 50 (50) |
| Megevand | 2019 | retrospective | 2010-2015 | 50 | 50 | 28/22 | 24/26 | - | - | - | - | 9 (18) | 7 (14) |
| Solaini | 2019 | retrospective | 2007-2017 | 305 | 84 | 162/143 | 39/45 | - | - | - | - | - | - |
| Chi-Yong Ngu | 2018 | retrospective | 2015-2017 | 16 | 16 | 10/6 | 6/10 | 68.6±10.9 | 69.6±9.6 | 23.7±3.8 | 24.7±4.2 | 8 (50) | 12 (75) |
| Spinoglio | 2018 | retrospective | 2005-2013 | 101 | 101 | 57/44 | 47/54 | 71.2±10.2 | 71.2±10.6 | 25.1±4 | 25.8±4.4 | 38 (37.6) | 43 (42.6) |
| Trastulli | 2015 | retrospective | 2005-2014 | 102 | 40 | 56/46 | 25/15 | 68.8±11.6 | 71.5±10.3 | 25.6±3.8 | 26.6±4 | 39 (38.2) | 14 (35) |

^a^Standard deviation

^b^Body Mass Index

^c^American Society of Anesthesiologists

^d^Robot-assisted intracorporeal anastomosis

^e^Laparoscopic intracorporeal anastomosis

Supplemental Table 3 Surgical outcomes of the study population

| First Author | Sample size | | Operative time (mean ± SD^a^) | | Blood loss  (mean ± SD) | | Open conversion (n (%)) | | Anastomotic leakage (n (%)) | | Surgical site infection (n (%)) | |
| --- | --- | --- | --- | --- | --- | --- | --- | --- | --- | --- | --- | --- |
|  | Rt-IA^b^ | Lap-IA^c^ | Rt-IA | Lap-IA | Rt-IA | Lap-IA | Rt-IA | Lap-IA | Rt-IA | Lap-IA | Rt-IA | Lap-IA |
| Serra-Aracil | 68 | 35 | 217.5±54.81 | 190±44.4 | - | - | 0 | 0 | 2 (3) | 2 (6) | 3 (4) | 4 (11) |
| Angelis | 149 | 149 | 191.12±51.54 | 179.19±62.19 | 65.04±45.06 | 73.27±60.63 | 3 (2) | 0 | 6 (4) | 7 (5) | 0 | 2 (1) |
| Ceccarelli | 20 | 20 | 225.2±73 | 165.9±30.2 | - | - | - | - | - | - | - | - |
| Migliore | 46 | 170 | 242.43±47.51 | 187.6±56.6 | - | - | 1 (2) | 6 (4) | - | - | - | - |
| Merola | 94 | 94 | 207.47±44.93 | 135.46±33.86 | - | - | 3 (3) | 0 | - | - | - | - |
| Blumberg | 21 | 101 | 330±100 | 212±66 | 100±58 | 100±153 | 0 | 5 (5) | - | - | - | - |
| Megevand | 50 | 50 | - | - | - | - | 0 | 7 (14) | - | - | - | - |
| Solaini | 305 | 84 | 253.5±24 | 162.5±17.5 | 50±0 | 56.25±6.25 | 3 (1) | 0 | 8 (3) | 3 (4) | 21 (7) | 6 (7) |
| Chi-Yong Ngu | 16 | 16 | 230±43.75 | 182.5±41.25 | - | - | - | - | - | - | - | - |
| Spinoglio | 101 | 101 | 279±80 | 236±68 | - | - | 0 | 7 (7) | 1 (1) | 1 (1) | 5 (5) | 10 (10) |
| Trastulli | 102 | 40 | 287.4±76.4 | 204.3±51.9 | 80±60 | 95±85 | 4 (4) | 6 (15) | 3 (3) | 0 | 5 (5) | 4 (10) |

^a^Standard deviation

^b^Robot-assisted intracorporeal anastomosis

^c^Laparoscopic intracorporeal anastomosis

Supplemental Table 4 Surgical outcomes of the study population

| First Author | Postoperative ileus (n (%)) | | Postoperative hemorrhage (n (%)) | | Time to first oral intake  (days (mean ± SD^a^)) | | Time to first flatus (days (mean ± SD)) | | Length of hospital stay (days (mean ± SD)) | |
| --- | --- | --- | --- | --- | --- | --- | --- | --- | --- | --- |
|  | Rt-IA^b^ | Lap-IA^c^ | Rt-IA | Lap-IA | Rt-IA | Lap-IA | Rt-IA | Lap-IA | Rt-IA | Lap-IA |
| Serra-Aracil | 4 (6) | 3 (9) | 5 (7) | 2 (6) | - | - | - | - | 5.8±2.6 | 13.2±25.5 |
| Angelis | 0 | 3 (2) | - | - | 3.01±1.83 | 3.34±1.46 | 2.07±1.12 | 2.24±1.11 | 7.54±7.99 | 8.29±4.87 |
| Ceccarelli | 2 (10) | 1 (5) | 0 | 0 | 3.2±1.2 | 3.5±1.1 | 3.2±1.2 | 3.0±1.2 | 7.2±1.6 | 7.8±3.0 |
| Migliore | - | - | - | - | - | - | 1.65±1.04 | 1.61±0.83 | 7.25±3.75 | 12.5±9.5 |
| Merola | - | - | - | - | - | - | 2.23±0.7 | 2.2±1.24 | 4±2 | 4±2 |
| Blumberg | - | - | - | - | - | - | - | - | 3±6.4 | 5±1.7 |
| Megevand | - | - | - | - | - | - | - | - | - | - |
| Solaini | - | - | 12 (4) | 6 (7) | 2.75±0.25 | 2.25±0.25 | 2.75±0.25 | 2.25±0.25 | 7.25±0.75 | 8±1 |
| Chi-Yong Ngu | - | - | - | - | - | - | - | - | 6±2.75 | 7±3.25 |
| Spinoglio | 0 | 1 (1) | 4 (4) | 2 (2) | 1.3±1.1 | 1.1±0.5 | 1.9±1 | 1.8±0.8 | 7.9±5.2 | 7.9±3.5 |
| Trastulli | 4 (4) | 1 (3) | 5 (5) | 1 (3) | - | - | 3.25±1.75 | 4±1.5 | 8.25±4.75 | 7±2.75 |

^a^Standard deviation

^b^Robot-assisted intracorporeal anastomosis

^c^Laparoscopic intracorporeal anastomosis

Supplemental Table 5 Quality assessment of included studies using the Newcastle-Ottawa Scale

| Study | Selection  (0-4) | Comparability  (0-2) | Outcome  (0-3) | Total  (0-9) |
| --- | --- | --- | --- | --- |
| Serra-Aracil et al. 2024 | 3 | 1 | 2 | 6 |
| de'Angelis et al. 2023 | 4 | 2 | 3 | 9 |
| Ceccarelli et al. 2021 | 3 | 2 | 3 | 8 |
| Migliore et al. 2021 | 3 | 2 | 3 | 8 |
| Merola et al. 2020 | 3 | 1 | 2 | 6 |
| Blumberg et al. 2019 | 2 | 1 | 2 | 5 |
| Mégevand et al. 2019 | 3 | 1 | 2 | 6 |
| Solaini et al. 2019 | 3 | 2 | 2 | 7 |
| Ngu et al. 2018 | 3 | 1 | 2 | 6 |
| Spinoglio et al. 2018 | 3 | 2 | 3 | 8 |
| Trastulli et al. 2015 | 3 | 1 | 2 | 6 |

**Supplemental Figure legends**

Fig. 1 The meta-analysis for time to first oral feeding

Fig. 2 The meta-analysis for time to first flatus

Fig. 3 The meta-analysis for length of hospital stay

Fig. 4 The meta-analysis for open conversion

Fig. 5 The meta-analysis for anastomotic leakage

Fig. 6 The meta-analysis for surgical site infection

Fig. 7 The meta-analysis for postoperative ileus

Fig. 8 The meta-analysis for postoperative hemorrhage

Supplemental Fig. 1

Supplemental Fig. 2

Supplemental Fig. 3

Supplemental Fig. 4

Supplemental Fig. 5

Supplemental Fig. 6

Supplemental Fig. 7

Supplemental Fig. 8

Supplementary Material

Detailed Search Strategy

A systematic literature search was performed to identify studies comparing robot-assisted intracorporeal anastomosis (Rt-IA) and laparoscopic intracorporeal anastomosis (Lap-IA) in colon resection.

The search was conducted in PubMed (MEDLINE), Web of Science, Scopus, and the Cochrane Library from database inception to December 2024. No restrictions were applied regarding publication date. Only articles published in English were included. Human studies were selected where applicable.

1. PubMed (MEDLINE)

   The search strategy incorporated both Medical Subject Headings (MeSH) and free-text terms:

   ("Colonic Neoplasms"[MeSH] OR "colon cancer" OR "colorectal cancer") AND
   ("Anastomosis, Surgical"[MeSH] OR "intracorporeal anastomosis" OR "IA") AND
   ("Robotics"[MeSH] OR "robotic surgery" OR "robot-assisted surgery") AND
   ("Laparoscopy"[MeSH] OR "laparoscopic surgery") AND
   ("Colectomy"[MeSH] OR "colon resection" OR "colectomy")

   Search fields: Title/Abstract and MeSH Terms
   Filters: Human studies, English language
2. Web of Science

   The following search strategy was used:

   TS=("intracorporeal anastomosis" OR "IA") AND
   TS=("robotic surgery" OR "robot-assisted surgery") AND
   TS=("laparoscopic surgery") AND
   TS=("colon resection" OR "colectomy")

TS=("colon cancer" OR "colorectal cancer")

Search field: Topic (Title, Abstract, Keywords)
Filters: English language

3. Scopus

Scopus was also searched using the following terms:

("intracorporeal anastomosis" OR "IA") AND ("robotic surgery" OR "robotic" OR "robot-assisted surgery") AND ("laparoscopic surgery" OR "laparoscopic") AND ("colon cancer" OR "colorectal cancer")

No additional eligible studies were identified.

4. Cochrane Library

The Cochrane Library was searched using the following terms:

"intracorporeal anastomosis" AND "robotic surgery" AND "laparoscopic surgery" AND "colectomy" AND "colon cancer"

Search field: Title, Abstract, Keywords
Filters: None

No additional eligible studies were identified through the Cochrane Library search.
